# Tuberculosis of the Nose, Mouth and Pharynx*Read before the Physicians’ Club, December 23, 1887.

**Published:** 1888-02

**Authors:** Frank Hamilton Potter

**Affiliations:** Lecturer on Laryngology, Medical Department, Niagara University; 42 West Tupper street


### *TUBERCULOSIS OF THE NOSE, MOUTH AND PHARYNX.**

By FRANK HAMILTON POTTER, M. D.,

Lecturer on Laryngology, Medical Department, Niagara University.

The following notes were made for my own use. Their purpose was to have, in a connected form, an account of the work accomplished in the study of the tuberculous processes, as seen in the nose, mouth and pharynx, especially with reference to their diagnosis and treatment. Particular attention is paid to what has been done on this subject during the last year. After they were well under way, it occurred to me that if they could be joined together so as to be presentable, and read before this club, it would be the means of introducing an important subject for discussion, and one worth the most serious consideration. There was also the hope that much of value would be gained through the added experience of others who perchance had paid some attention to the subject.

We will first consider the manifestations of tuberculosis as seen in the nasal passages. It is only recently that this part of

* Read before the Physicians' Club, December 23, 1887.

the subject has received any attention whatever. It was thought that tuberculosis did not attack the nasal passages; and Fraenkel, but a few years ago, in volume iv. of Ziemssen's Cyclopedia, in considering ulcerations of the nasal mucous membrane, remarks that "Tubercular (phthisical) ulcers of the nose seem to be among the greatest rarities. I, myself, have never seen anything like them, and find only one allusion to tuberculosis of the nose in literature." And he quotes Willigk as stating that in the Institute of Pathological Anatomy, at Prague, tuberculosis of the nasal septum was only encountered once in 476 tubercular corpses. Since this was written, it has been shown that tubercular nasal disease is not so rare as Fraenkel supposed. Cases have been reported showing various forms of the disease by Sokolowski, by Volkmann, by Koenig and Riedel, by Cluttar, by Schäffer, and Nasse, and by Cartaz. From these observers, it appears that the disease occurs here under two forms: in the first as an ulceration, and in the second as a neoplasm. Both forms may be seen in the same case and at the same time. "The ulcer has a diameter of one to two centimetres, and a form more or less rounded. The situation is usually upon the septum, a little distant from the nasal meatus. The base of the ulcer is a pale reddish gray, and covered with muco-pus; caseous masses are seen embedded on certain of its aufractuosities, or fine grey granulations are seen in relief. The edges are sometimes projecting, sometimes forming, sometimes a light grey ring, sometimes sharply cut and toothed with small excavations. There is little pain, or interference with nasal respiration."

In the second form, the neoplasm appears usually as a tumor. It varies in size from a small pea to a fairly large nut. In one case, it developed as a large fibroma of the septum. In all the reports I have seen, the tumor was found upon the septum, though Cartaz speaks as if it could be found in other places. Associated with these tumors, there is usually coryza, and nasal respiration is interfered with, especially if the growths are large and the coryza severe. They bleed easily, and are harder in the center than upon the outer portions. The diagnosis before removal is difficult, unless there are other signs of tuberculosis present, though a hereditary predisposition to the disease should put one on guard about them. Tubercle bacilli are found in them, but they are not abundant. After removal, there is an inclination to recurrence and to ulceration, which may involve the bony parts and cause perforation of the septum. It is proper to notice that Dr. Bresgen, of Frankfort, considers the tumors with the above characteristics as lupus, and not tuberculosis. This seems to be an unnecessary refinement, and tends to confuse, for lupus itself is a tuberculous process, and the treatment of the former must be based upon the recognition of its pathological relationship to the latter.

The local treatment of these conditions would naturally follow here, but, in order to prevent repetition, we will postpone its consideration to the end of the paper. In the pharynx and mouth, we find that the tubercular processes appear more frequently than in the nose, but, on the other hand, less frequently than in the larynx and lungs. It seems that the deeper we descend in the air passages, the more often is this dreadful disease encountered. Seen in the mouth or pharynx, the local manifestations take the form of ulcers generally, but Ariza reports a granular condition also; and Volkmann says that small tumors, showing a tendency to soften in the centers, may be found. The latter manifestations are rare. The most complete description of the ulcers is that of Mr. Henry T. Butlin, in his work "On the Diseases of the Tongue." As it applies equally well to the ulcers found in the other parts of the mouth and pharynx, I quote it entire: "A completely-developed tuberculous ulcer, not too broken down and sloughy, presents most of the following characters: The surface is uneven, pale and rather flabby, granulated, or often covered with yellowish-grey, viscid or coagulated mucus; the edges are sometimes sharp-cut, sometimes beveled, seldom elevated, or everted, or undermined; not usually very red, but often redder than the surrounding tongue; there is very little surrounding induration: indeed, there may be none; the adjacent portions of the tongue are generally a little swollen, sometimes decidedly swollen and sodden; the outline of the ulcer has no characteristic shape, but the borders are often sinuous, and the shape is not unusually oval, or ovoid, or elongated. In the immediate neighborhood of the ulcer, and perhaps extending for some distance beyond it, are sometimes observed tiny yellowish-grey points, or patches, or elevations, or, in the place of these, minute ulcers, which in time increase in size. The depth of the ulcer varies much: in the earlier stages it is superficial, but, as the disease advances, it may eat deeply into the substance of the tongue, and, eating more deeply at one part than another, may present different depths at different parts. It is almost invariably painful in the latter stages, and there is almost always salivation. In this description, some resemblance may be discovered to the tuberculous ulcers of other parts of the body; in the pale and flabby granulations, the sharp cut or beveled edges, the absence of surrounding inflammation and induration."

It is next important to know that these ulcers may appear in any part of the mouth or pharynx, but there are some favorable situations where they are found with greater frequency. According to the investigations of Dr. Delavan, these situations are as follows, and in the order named: Tongue, pharynx, gums, velum palati, tonsils and cheeks; nearly one-half appearing on the tongue. In this list, the lips are not mentioned, and it is, therefore, proper to say that Volkmann and Hausemann report such cases, and a most instructive example has recently been reported in a Vienna medical journal. This case will be considered more at length under the head of the infectiousness of these ulcers.

It used to be held that these local lesions were always a sign ot the constitutional disease, but one can no longer believe this. There are many well-authenticated cases in which the local trouble preceded the general disease, and others where the local was the only manifestation present, and which disappeared after a time under appropriate treatment. In the seven cases of buccal tuberculosis reported by Dr. Delavan in 1886, three were of primary; and in the twenty-four cases where the disease was located on the tongue, referred to by him in the same paper, nine were primary. We can thus see how important this subject is, and how carefully these cases should be considered. Especially is this so, as the primary lesion is often—as we shall see further on—susceptible of successful treatment.

Tuberculosis of these regions, as of other regions, may be either acute or chronic; and the acute form, contrary to expectation, need not necessarily lead to a fatal result. As the description of the lesions given above apply rather to the chronic variety, we will give a few words to a case of acute tubercular pharyngitis reported by Ariza. The patient was a physician, delicate, aphonic, and suffering from the greatest odynphagia. Several small red outgrowths, springing from an ulcerated base, were located on the anterior pillars of the fauces, while in the pharynx were two or three whitish granules of the size of a pin's head, and another on the base of the uvula. None of these granulations had the characteristic yellow color. The patient had experienced three attacks of sore throat within a few months, but described himself as being perfectly well since the last. Tubercle-bacilli, of recent origin, were abundant. The patient recovered under appropriate treatment. The points of interest in regard to these cases, as suggested by the author, are (1) that they are rare; (2) that the appearance of the lesions in different localities may not always correspond in point of time to the classical descriptions that have been given; and (3) that they are curable.

Another interesting point concerning these lesions is, whether or not they are contagious—that is, whether tuberculosis can be transmitted through them by actual contact. In the light of modern pathology, there does not seem any good reason to doubt that the disease can be transmitted in this way. We must consider that tuberculosis is a bacillary disease, and that both its local and general manifestations are due to the growth and development of these bacilli. It is certainly conceivable that the bacilli from an ulcer could be transmitted and find lodgment on any other abraded mucous surface coming in contact with it.

In this way, we could have auto-infection, or the transmission of the disease from one individual to another.

A case reported in the *Wiener Med. Bl.*, November 8, 1886, may be cited as confirmatory of this position: A man suffered from tuberculosis of the lungs, and, as usual, succumbed ultimately to this affection. A tubercular ulcer on his gum proved the medium of propagation of the affection from husband to wife. A few years after the man's death, the widow noted the appearance of an ulcer on her lips, which proved to be of a tuberculous nature. Direct infection through kissing was, in this instance, the evident source of this ulcer. After all therapeutic interferences (including cauterization with lactic acid) had proven useless, the ulcer was cauterized with the Pacquelin cautery, and disappeared. * It is, therefore, most important that any ulcer seen anywhere in these regions should be carefully diagnosticated. The general description of these tubercular ulcers has been given above, and, if this be carefully considered, much will be done towards a correct diagnosis. Especially if there is present any evidence of general tuberculosis, the nature of these ulcers can be arrived at with great certainty. The only sure proof, however, is the finding of the bacillus tuberculosis in the ulcer or its discharges. For this purpose, the ulcer should be scraped, and the scrapings given to a competent pathologist for investigation. If the bacillus is found, all doubt as to the nature of the lesion is cleared up at once.

The possibilities of a diagnostic error are associated with the diseases, carcinoma and syphilis, as they are seen in these regions. In this regard, Hausemann remarks that it matters little to the patient whether the extirpated growth is of a carcinomatous or of a lupus, tubercular nature, but, if syphilitic, perverted therapeutics will probably make mischief. I think we may take the liberty, in passing, to doubt the first half of this statement, especially in view of the successes reported during the last year in the treatment of local tuberculosis.

Before considering the treatment of these conditions, one more point of great interest should be mentioned, viz., the

* Abstract of *Therapeutic Gazette*, May, 1886.

extreme youth of some of the subjects. Thus Laurent reports the case of tubercular disease of the tongue in a child, eleven years of age; Delavan, in the report already referred to, mentions a case of primary tubercular ulcer of the cheek in a child, aged six; and Clutton records and illustrates a unique case of tubercular ulceration of the hard and soft palate and larynx in a girl, aged fifteen, in which partial healing took place. *

The local treatment—which is alone considered in this paper—naturally divides itself into measures that are medical or surgical. The medical measures consist, in the first place, in keeping the parts clean, and then in applying directly to the diseased tissue those agents found to have a beneficial effect on tuberculous processes. The cleaning must be done carefully, so that all discharges are removed. This can be done by dissolving them in an alkaline solution, and wiping them away with absorbent cotton. If they resist removal by these simple means, forcible measures should be used, as scraping with the curette, etc. Our remedies are limited, consisting of cocaine, corrosive sublimate, iodoform, iodol, lactic acid and menthol.

The use of cocaine is familiar to all. Corrosive sublimate is a dangerous remedy to use, though it is effective. Iodoform is objectionable on account of its disagreeable odor. Iodol is practically without odor or taste. It contains seven per cent. of iodine, and parts with it much more rapidly than iodoform. It can be used freely, with but little danger of toxic effects. "It is antiseptic and anæsthetic, a promoter of granulation and healing, arrests suppuration, and deodorizes foul secretions." †

Tuberculous ulcers have been reported to heal perfectly under daily applications of iodol by Lublinski, Wolfeuden and others. On the whole, it is one of our most efficient agents. Lactic acid has proved disappointing. It is painful to apply even under cocaine. It acts best where there is a good deal of induration surrounding the lesion. It is applied by saturating a piece of absorbent cotton with it, and pressing it against the part.

---

* *Journal of Laryngology, etc.*, March, 1887; page 92.

† *Journal of Laryngology*, July, 1887.

A good deal can be said in favor of menthol. It is slightly analgesic, and agreeable in its application. It is highly destructive of the bacillus tuberculosis, according to the testimony of Koch, Sormain and Bagnatelli. As far as known, it produces no toxic symptoms, and can, therefore, be used freely. It is very soluble in alcohol and the various oils. I have used it dissolved in the oleum petrolina in the strength of five to thirty per cent. As I have only used it in cases of laryngeal and pulmonary tuberculosis, a report of my observations would be beyond the scope of this paper. It may not be improper, however, to say that my experience with its use confirms the opinions of Rosenberg, Hyndman and others; that it possesses a most favorable influence over the tubercular lesions. It certainly deserves an extended trial.

There are many localities, as, for instance, the tongue, where the medical measures mentioned above will fail, because the applications are immediately washed away by the natural secretions of the parts, thus preventing any prolonged contact of the medicine with the diseased tissue. In these cases, we must resort to surgical measures. These consist in scraping the ulcer with the sharp curette, in excising it with the knife or the pacquelin, or the galvano-cautery, and then in promoting healing by the means detailed above. Excision seems to be followed by the best results, for the reason that the ulcers are often irregular, and it is impossible to remove all the diseased tissue by scraping. When you have to deal with a neoplasm that is large, it can be removed with the snare, and its base cauterized with chromic acid or the galvano-cautery; or, if the growth is small, it can be removed by the galvano-cautery. The details of these operations need not be given here. It is more a question of applying the surgical relief to locations that are often very difficult to reach than one involving any principle of operation not already well understood.

In conclusion, I trust enough has been said to quicken the interest in these local manifestations of tuberculosis, so that, when met, a proper diagnosis may be made and a proper treatment begun.

42 West Tupper street.

